# RNA Structural Requirements for Nucleocapsid Protein-Mediated Extended Dimer Formation

**DOI:** 10.3390/v14030606

**Published:** 2022-03-15

**Authors:** Françoise Chaminade, Jean-Luc Darlix, Philippe Fossé

**Affiliations:** 1 LBPA, UMR8113 CNRS, ENS Paris-Saclay, Université Paris-Saclay, 91190 Gif-sur-Yvette, France; chaminadefrancoise@gmail.com; 2 Laboratoire de Bioimagerie et Pathologies, UMR7021 CNRS, Faculté de Pharmacie, Université de Strasbourg, 67400 Illkirch, France; jldarlix@gmail.com

**Keywords:** nucleocapsid protein, RNA dimerization, Rous sarcoma virus (RSV), HIV-1, retrovirus, RNA secondary structure

## Abstract

Retroviruses package two copies of their genomic RNA (gRNA) as non-covalently linked dimers. Many studies suggest that the retroviral nucleocapsid protein (NC) plays an important role in gRNA dimerization. The upper part of the L3 RNA stem-loop in the 5′ leader of the avian leukosis virus (ALV) is converted to the extended dimer by ALV NC. The L3 hairpin contains three stems and two internal loops. To investigate the roles of internal loops and stems in the NC-mediated extended dimer formation, we performed site-directed mutagenesis, gel electrophoresis, and analysis of thermostability of dimeric RNAs. We showed that the internal loops are necessary for efficient extended dimer formation. Destabilization of the lower stem of L3 is necessary for RNA dimerization, although it is not involved in the linkage structure of the extended dimer. We found that NCs from ALV, human immunodeficiency virus type 1 (HIV-1), and Moloney murine leukemia virus (M-MuLV) cannot promote the formation of the extended dimer when the apical stem contains ten consecutive base pairs. Five base pairs correspond to the maximum length for efficient L3 dimerization induced by the three NCs. L3 dimerization was less efficient with M-MuLV NC than with ALV NC and HIV-1 NC.

## 1. Introduction

A hallmark of retroviruses is that they package two copies of their genomic RNA (gRNA) to form an infectious virion. The two packaged gRNA molecules interact to generate an RNA dimer in which the subunits are held together by noncovalent bonds. Conservation of the dimeric gRNA among retroviruses is required for several stages of the retroviral life cycle (reviewed in Ref. [1]). Interestingly, dimerization of two non-identical copies of gRNA increases the genetic diversity by facilitating recombination events during reverse transcription [2].

After virus budding, the non-infectious immature form of the virus particle is reorganized into the infectious mature form [3]. The dimeric gRNA isolated from immature virus particles is less stable than that isolated from mature virus particles (virions) [4,5,6]. The processes of maturation of the viral particle and stabilization of the dimeric gRNA require the cleavage of the Gag polyprotein by the viral protease [5,6,7,8,9]. Results obtained with Gag mutants suggest that the nucleocapsid protein (NC) is the Gag cleavage product that is primarily responsible for dimeric gRNA stabilization [5,9,10,11]. Consistent with this notion, NCs from different retroviruses promote viral RNA dimerization in vitro [12,13,14].

There are numerous studies on dimerization of human immunodeficiency virus type 1 (HIV-1) gRNA (see Ref. [1] and references therein). RNA transcripts containing the 5′-end of the HIV-1 genome can form low- and high-stability dimers in vitro that are named loose and tight dimers, respectively [15,16]. In the absence of HIV-1 NC (NCp7) under physiological conditions, HIV-1 RNAs form loose dimers through the dimerization initiation site (DIS) corresponding to stem-loop 1 (SL1) [16,17,18,19]. The intermolecular base pairing in HIV-1 loose dimers is a loop–loop interaction involving the apical loop of SL1 [16,18,19]. NCp7 can promote the formation of tight HIV-1 RNA dimers via SL1 [20,21]. Several studies support a model in which the intermolecular base pairing in HIV-1 tight dimers is an extended duplex involving the whole SL1 sequence (35 nt) [16,22,23,24,25]. The secondary structure of SL1 includes two stems separated by one internal loop. NCp7-mediated SL1 dimerization depends on the internal loop and the length of stems [26,27]. This observation is not surprising because destabilization of stems is necessary for extended dimer formation.

**Figure 1:**
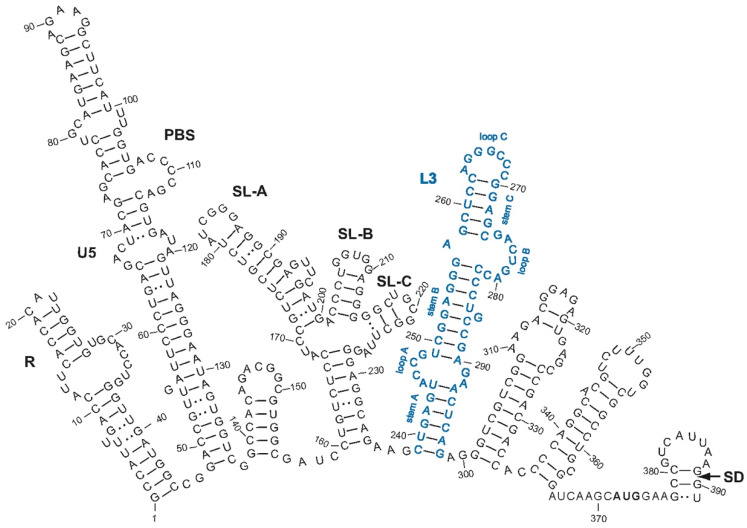
Predicted secondary structures for the ALV 5′-leader RNA [30]. Numbering is relative to the genomic RNA cap site (+1). R, repeated sequence; U5, unique sequence in 5′; PBS, primer binding site; SL-A, B, and C, stem-loop structures of the minimal packaging signal [31]; L3 in blue font, stem-loop structure involved in ALV gRNA dimerization; the structural elements of L3 are indicated in blue; AUG in bold, *gag* initiation codon; SD, splice donor site.

In retroviruses other than HIV-1, it is not well known how the RNA structures modulate the efficiency of NC-mediated RNA dimerization. The nucleocapsid protein of the alpharetroviruses (NCp12) is necessary for gRNA dimer formation in the context of the virion [10]. NCp12 promotes dimerization of RNA transcripts containing the 5′-end of the Rous sarcoma virus (RSV) genome [12]. The 208–270 sequence plays an essential role in the NCp12-mediated RSV RNA dimerization process [12]. This sequence located in the 5′-leader contains part of the L3 stem-loop (Figure 1) that is responsible for loose dimerization of avian leukosis virus (ALV) RNAs [28,29].

We showed that the dimerization properties defined for our ALV strain (type Schmidt-Ruppin A) differ from other alpharetrovirus strains [29]. More precisely, RSV (strain Prague C) RNA dimerization in vitro requires the L3 and SL-A stem-loop structures [32]. In agreement with the role of L3 in RNA dimerization, deletion of this stem-loop drastically decreases RSV (type Schmidt-Ruppin A) replication [33]. A recent ex vivo study supports the notion that dimerization of RSV (type Schmidt-Ruppin A) gRNA within the infected cell depends on the presence of L3 [34].

**Figure 2:**
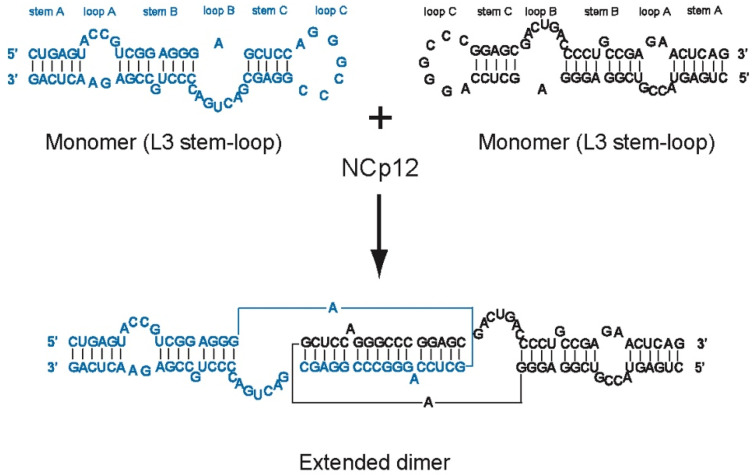
Secondary structures for the L3 stem-loop in the monomer and the extended dimer.

In a previous study [35], we investigated the role of L3 in NCp12-mediated dimerization of ALV RNA. We showed that NCp12 promotes the formation of tight ALV RNA dimers via L3. We also showed that the intermolecular base pairing in tight dimers is an extended duplex involving only the upper part of the L3 stem-loop (Figure 2). Therefore, the two subunits in ALV RNA tight dimers are not held together by the whole L3 sequence. In this study, we investigated the roles of stems and internal loops in the NCp12-mediated L3 dimerization process. We found that loop B (Figure 2), but not its sequence, is required for efficient extended RNA dimer formation. Our results show that the length of stems modulates the efficiency of NCp12-mediated L3 dimerization. We also found that L3 dimerization induced by NCs from HIV-1 and Moloney murine leukemia virus (M-MuLV) depends on the length of stem C (Figure 2).

## 2. Materials and Methods

### 2.1. Protein Preparation

NCp7 (NC(1–55)) of the HIV-1 MAL isolate, NCp10 of M-MuLV, and NCp12 of ALV (type Schmidt-Ruppin A) were synthesized by the Fmoc/opfp chemical method and purified to homogeneity by HPLC, as described previously [35,36,37,38].

### 2.2. Oligonucleotides

DNA oligonucleotides were purchased from Eurogentec (Liège, Belgique). Nucleotides of the avian leukosis virus of subgroup A (ALV-RSA termed ALV in the text) are numbered according to the Pr-C strain of RSV [39]. In the following oligonucleotide sequences, the upper-case letters indicate the bases that are complementary to the DNA sequence of ALV plasmid used for PCR, and the positions of the ALV sequence targeted by the oligonucleotides are in parentheses: O1, 5′-gtattcGAGCTCCAGGGCCCGGAGCaACTaACCCCTGCCGAG-3′ (256–291); O2, 5′-gtattcGAGCTctCCAGGGCCCGGagAGCGACTGACCC-3′ (256–282); O3, 5′-gtattcGAGCTctcgtCCAGGGCCCGGacgagAGCGACTGACCC-3′ (256–282); O4, 5′-gtattcGAGCTCCAGGGCCCGGAGCCCCTGCCGAGAAC-3′ (256–294); O5, 5’-gtattcGAGCTCCAGGGCCCGGAGCaAaaaAaCCCTGCCGAGAACTC-3′ (256–296); O6, 5′-gtattcGAGCTCaCAGGGCCCGtGAGCGACTGACCCC-3′ (256–284); O7, 5′-gtattcGAGCTCgCAGGGCCCGcGAGCGACTGACCCC-3′ (256–284); O8, 5′-gtaatcGGGCCCGGAGCGACTGACCCCTCCGAcGACTCAGAGGGTCG-3′ (264–305); O9, 5′-gtaatCGGGCCCTGGAGCTCCCTCCGACGACTCTATAGTGAGTC-3′ (270–241); OR1, 5′-gctctagacgctagCTCTCGAGCCGCC-3′ (629–617); OR2, 5′-ctcggcgcacgGCTAGCTCTCGAGC-3′ (629–621).

### 2.3. Construction of Plasmids

Standard molecular biology methods were used for plasmid construction. Restriction endonucleases and T4 DNA ligase were purchased from New England Biolabs (Evry-Courcouronnes, France). The expand high-fidelity PCR system was from Roche Diagnostics (Meylan, France). Cloned sequences and mutations were verified by DNA sequencing. Plasmid pEP241 contains the 241–629 sequence of the ALV genome [29]. The plasmid mutants differ from pEP241 by base substitutions and deletions in the 245–292 sequence. Plasmids pFCLm1LB, pMBL+2, pMBL+5, and pMBLdLB were generated by PCR amplification of linearized pEP241 with EcoRI using the pairs of oligonucleotides O1/OR1, O2/OR1, O3/OR1, and O4/OR1, respectively. The resulting PCR products were digested with SacI and XhoI and ligated into pEP241 digested with the same enzymes. Plasmids PFCLm2LB, PFCLAU+1, and PFCLGC+1 were generated by PCR amplification of linearized pIKS6A [35] with EcoRI using the pairs of oligonucleotides O5/OR2, O6/OR2, and O7/OR2, respectively. The resulting PCR products were digested with SacI and XhoI and ligated into pEP241 digested with the same enzymes. Plasmid pFCLdLA was generated by PCR amplification of linearized pIKdA263 [35] with Bsp120I using the pair of oligonucleotides O8/O9. The final PCR product was then digested with Bsp120I and intramolecularly ligated.

### 2.4. Synthesis, Labeling, and Purification of L RNAs

L RNAs were synthesized by in vitro transcription using the T7 RiboMAX^TM^ large-scale RNA production system (Promega, Charbonnières, France) and five micrograms of plasmids cleaved by the endonuclease restriction enzyme DdeI. The 5′- and 3′-ends of L RNAs correspond only to the ALV sequence, i.e., they do not possess additional sequences derived from the plasmid sequence. Each L RNA was purified by denaturing polyacrylamide gel electrophoresis as described previously [29]. Calf intestinal alkaline phosphatase (Roche Diagnostics, Meylan, France) was used to catalyze the dephosphorylation of the 5′-end of L RNAs. T4 polynucleotide kinase (New England Biolabs, Evry-Courcouronnes, France) and [γ-^32^P] ATP (PerkinElmer, Villebon-sur-Yvette, France) were used to label the 5′-end of L RNAs. Each L RNA labeled at its 5′-end was purified by denaturing polyacrylamide gel electrophoresis.

### 2.5. RNA Dimerization Assay

To prepare the heat-denatured L RNAs (lanes D in the gels), 1.9 pmol of each L RNA in 10 µL of double-distilled water was incubated at 90 °C for 2 min and placed on ice for 2 min and mixed with 3.5 µL of loading buffer (50% *w/v* glycerol, 0.05% *w/v* bromophenol blue, 0.05% *w/v* xylene cyanol). The dimerization assay was performed in the final volume of ten microliters and the final concentrations of 20 mM Tris-HCl, pH 7.5, 50 mM NaCl, 0.2 mM MgCl_2_, and 5 mM DTT [35]. Each labeled L RNA (1.9 pmol at 10^4^ cpm/pmol) in 6 µL of water was incubated at 90 °C for 2 min and placed on ice for 2 min. The NC buffer was added, and each sample was incubated at 37 °C for 15 min in the absence or presence of the nucleocapsid protein (NCp7, NCp10, or NCp12) at various concentrations. At the end of incubation, 2 µL of SDS-EDTA solution (7.1% SDS and 39.6 mM EDTA) was added to the sample. Then, RNA was phenol–chloroform extracted, and the aqueous phase was mixed with 3.5 µL of loading buffer. The samples were analyzed by electrophoresis on a 12% poylyacrylamide gel (19:1 (*w/v*), acrylamide/bisacrylamide) at 25 °C in the TBE buffer (89 mM Tris-borate (pH 8.3), 2 mM EDTA). After electrophoresis, the gel was fixed, dried, and autoradiographed. The monomeric (m) and dimeric (d) forms of L RNAs were quantified using a Typhoon^TM^ TRIO (GE Healthcare, Buc, France) and ImageQuant software (GE Healthcare, Buc, France). The percent of dimer was determined as 100 × (d/(m + d)).

### 2.6. Analysis of the Thermal Stability of the LdLB Dimer

Dimerization was performed in the final volume of 80 µL and the final concentrations of 20 mM Tris-HCl, pH 7.5, 50 mM NaCl, 0.2 mM MgCl_2_, and 5 mM DTT. Labeled LdLB RNA (15.2 pmol at 10^4^ cpm/pmol) was dissolved in 48 µL of water, heated at 90 °C for 5 min, and placed on ice for 5 min. The NC buffer was added, and the sample was incubated with NCp12 (866 pmol) at 37 °C for 15 min. Sixteen microliters of the SDS-EDTA solution (7.1% *w*/*v* SDS and 39,6 mM EDTA) were added to the sample before the phenol–chloroform extraction. Aliquots (10 µL) of the aqueous phase were heated for 5 min at temperatures ranging from 30 to 75 °C before the addition of 3.5 µL of loading buffer. Aliquots were analyzed by 12% polyacrylamide gel electrophoresis as described above.

## 3. Results

### 3.1. Design and Analysis of Mutant RNAs

**Figure 3:**
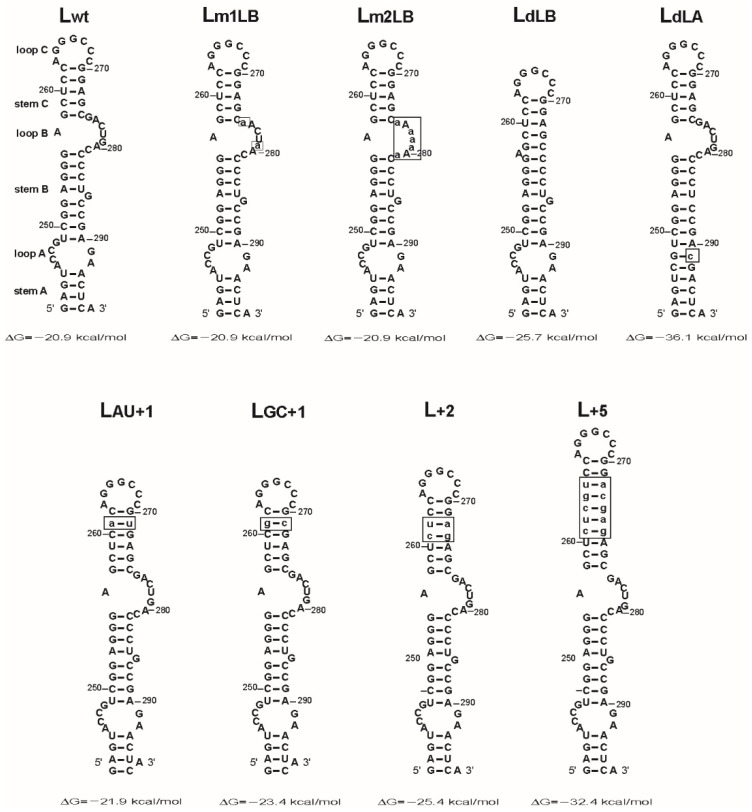
Predicted secondary structures for the L3 sequence of LRNAs containing mutations or deletions. The mfold program [40] predicted the most stable secondary structure for each RNA. Numbering is relative to the genomic RNA cap site (+1). The lower-case letters in the boxes indicate the mutations.

Nine RNAs corresponding to the wild-type and mutant conformations of the L3 domain were generated by in vitro transcription (Figure 3). The mfold program predicts that the wild-type and mutant RNAs form the L3 stem-loop structure. In addition, the mutants preserve the intermolecular base pairing potential of the loop C and stem C sequences. Lm1LB and Lm2LB RNAs differ from the wild-type RNA by base substitutions in loop B. These mutants allowed us to investigate the role of the loop B sequence in the NCp12-mediated RNA dimerization process. LdLA and LdLB RNAs are deletion mutants that allowed us to study the destabilizing effect of loops A and B on the L3 stem-loop structure. LdLB RNA is deleted from loop B but conserves the A bulge between the stems B and C. LdLA RNA does not possess loop A and the G bulge. Stem C of LAU+1, LGC+1, L+2, and L+5 RNAs is extended by one, two, or five base pairs. To examine the effect of mutations on extended RNA dimer formation, we used conditions described in Materials and Methods, where we removed NCp12 before analysis by gel electrophoresis. RNAs were analyzed by native polyacrylamide gel electrophoresis at 25 °C in Tris-borate/EDTA buffer. The extended dimer, but not the loop–loop dimer, can be seen under these electrophoretic conditions [29,35].

### 3.2. Role of Loop B in NCp12-Mediated RNA Dimerization

**Figure 4:**
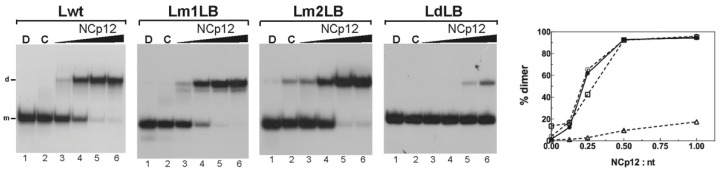
Dimerization of L RNAs harboring mutations in loop B. The 5′-end-labeled L RNAs in the absence (lanes C) or the presence of NCp12 (lanes 3–6) were incubated at 37 °C as described in Materials and Methods. After phenol–chloroform extraction, the samples were analyzed by electrophoresis on 12% polyacrylamide gels at 25 °C in the TBE buffer. Heat-denatured L RNAs (lanes D) were used to identify the bands corresponding to monomeric L RNAs. Lanes 3–6, protein to nucleotide molar ratios were 1:8, 1:4, 1:2, and 1:1. The monomeric and dimeric forms of L RNAs are indicated by m and d, respectively. The graph was derived from the gels shown in this figure. Filled circles, Lwt RNA; open circles, Lm1LB RNA; open squares, Lm2LB RNA; open triangles, LdLB RNA.

Specific interactions between NCp12 and the loop B sequence could play a role in NCp12-mediated L3 dimerization. Lwt RNA and the loop B mutants without NCp12 mainly remained monomeric after the treatments that removed the protein (Figure 4, lanes C). In the presence of increasing amounts of NCp12, a new band appeared that migrated at a rate expected for the dimeric RNAs (Figure 4, lanes 3–6). The replacement of two guanine residues in loop B with two adenine residues did not reduce RNA dimerization (Figure 4, Lm1LB). Similarly, the replacement of the GACUGAC sequence with the AAAAAAA sequence did not change the dimerization yield (Figure 4, Lm2LB). In contrast, the dimerization yield was decreased strongly by deleting loop B (Figure 4, LdLB). Therefore, loop B, but not its sequence, is required for efficient extended RNA dimer formation.

**Figure 5:**
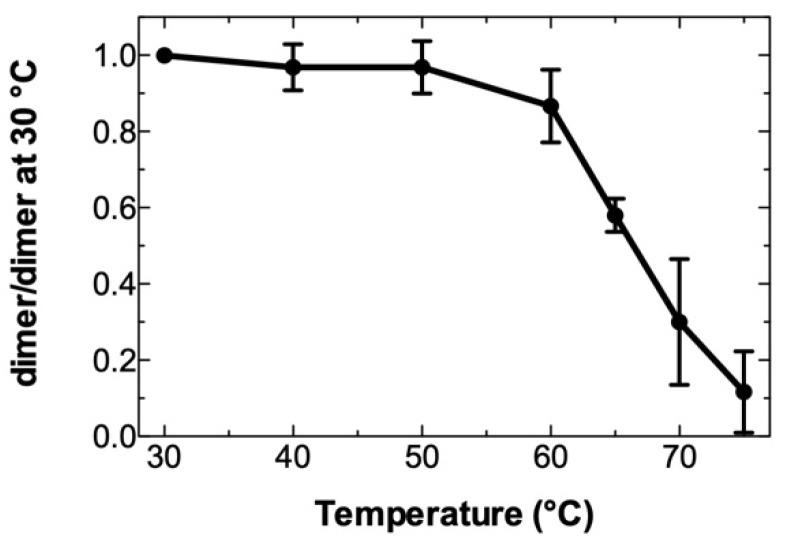
Thermal stability of the dimeric LdLB RNA. The melting curve was determined as described in Materials and Methods. Data are normalized according to the percentage of dimer at 30 °C and result from three experiments. Error bars show standard deviations.

Deletion of loop B leads to the formation of the long stem containing two bulges (Figure 3, LdLB). LdLB RNA could form an extended dimer, which would be longer than that of the wild type. Therefore, the LdLB extended dimer should be more thermostable than the wild-type extended dimer. To test this hypothesis, we determined the thermostability of the dimeric LdLB RNA (Figure 5). The Tm value of the LdLB dimer was about 66 °C, a value, which is significantly higher than the Tm of the wild-type dimer (53 °C) that has been determined under the same experimental conditions [35]. These results support the notion that the intermolecular base pairing in the LdLB extended dimer is not restricted to the loop C and stem C sequences.

### 3.3. Role of Loop A and the G Bulge in NCp12-Mediated RNA Dimerization

**Figure 6:**
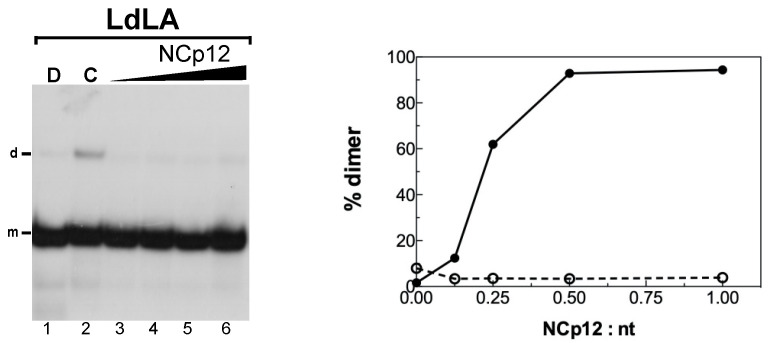
Deletion of loop A and the G bulge prevents NCp12-mediated L3 RNA dimerization. The 5′-end-labeled LdLA RNA in the absence (lane C) or the presence of NCp12 (lanes 3–6) was incubated at 37 °C as described in Materials and Methods. After phenol–chloroform extraction, the samples were analyzed by electrophoresis on a 12% polyacrylamide gel at 25 °C in the TBE buffer. Heat-denatured LdLA RNA (lane D) was used to identify the band corresponding to monomeric LdLA RNA. Lanes 3–6, protein to nucleotide molar ratios were 1:8, 1:4, 1:2, and 1:1. The monomeric and dimeric forms of LdLA RNA are indicated by m and d, respectively. The graph was derived from the gels shown in this figure and Figure 4. Filled circles, Lwt; open circles, LdLA.

NCp12 was unable to promote dimerization of LdLA RNA (Figure 6). This result shows that loop A and the G bulge are required for extended RNA dimer formation, i.e., the intermolecular base pairing of the loop C and stem C sequences is not possible in the absence of these two destabilizing elements. Deletion of these elements allows the formation of the long stem containing 14 base pairs without a bulge interruption (Figure 3, LdLA). NCp12 likely does not induce LdLA RNA dimerization because it cannot destabilize the long stem. 

### 3.4. Effect of the Length of Stem C on NCp12-Mediated RNA Dimerization

**Figure 7:**
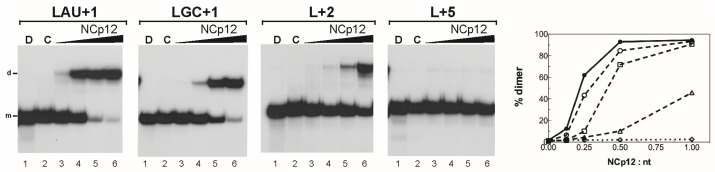
Influence of stem C extensions on NCp12-mediated L3 RNA dimerization. The 5′-end-labeled L RNAs in the absence (lanes C) or the presence of NCp12 (lanes 3–6) were incubated at 37 °C as described in Materials and Methods. After phenol–chloroform extraction, the samples were analyzed by electrophoresis on 12% polyacrylamide gels at 25 °C in the TBE buffer. Heat-denatured L RNAs (lanes D) were used to identify the bands corresponding to monomeric L RNAs. Lanes 3–6, protein to nucleotide molar ratios were 1:8, 1:4, 1:2, and 1:1. The monomeric and dimeric forms of L RNAs are indicated by m and d, respectively. The graph was derived from the gels shown in this figure and Figure 4. Filled circles, Lwt RNA; open circles, LAU+1 RNA; open squares, LGC+1 RNA; open triangles, L+2 RNA; open diamonds, L+5 RNA.

The results obtained with the LdLB and LdLA mutants suggest that the stability of stems A, B, and C modulates the efficiency of NCp12-mediated L3 RNA dimerization. Phylogenetic analysis of 28 alpharetrovirus sequences predicted that stem C contained 6 base pairs in one strain and 5 base pairs in 27 strains [28]. Conservation of a short stem C could be required for extended dimer formation. Stem C was extended by one, two, or five base pairs in the LAU+1, LGC+1, L+2, and L+5 mutants (Figure 3). The extension of stem C by the A:U base pair slightly decreased the dimerization yield at an NCp12 to nucleotide molar ratio lower than 1:1 (Figure 7, LAU+1). The extension of stem C by the G:C base pair significantly decreased the dimerization yield at an NCp12 to nucleotide molar ratio lower than 1:2 (Figure 7, LGC+1). The addition of two base pairs in stem C drastically reduced L3 stem-loop dimerization (Figure 7, L+2). Interestingly, NCp12 did not promote extended dimer formation when stem C was extended by five base pairs (Figure 7, L+5). Taken together, these results show that a short stem C (five to six base pairs) is required for efficient extended dimer formation. In addition, our results suggest that NCp12 can destabilize only short RNA stems flanked by loops, bulges, mismatches, or duplex ends.

### 3.5. Effects of NCp7 and NCp10 on L3 RNA Dimerization

**Figure 8:**
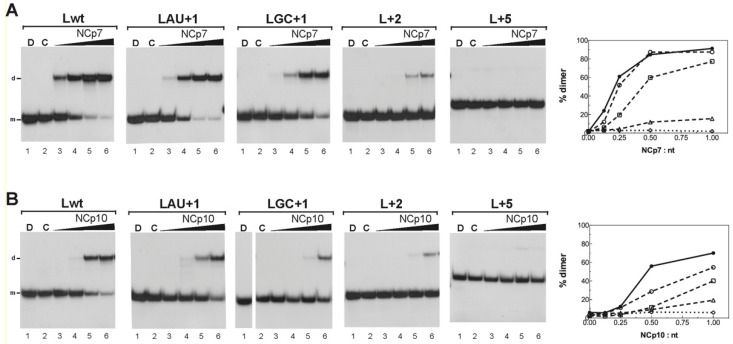
Influence of stem C extensions on L3 RNA dimerization induced by NCp7 and NCp10. (**A**) NCp7-mediated L3 RNA dimerization. (**B**) NCp10-mediated L3 RNA dimerization. The 5′-end-labeled L RNAs in the absence (lanes C) or the presence of NCp7 or NCp10 (lanes 3–6) were incubated at 37 °C as described in Materials and Methods. After phenol–chloroform extraction, the samples were analyzed by electrophoresis on 12% polyacrylamide gels at 25 °C in the TBE buffer. Heat-denatured L RNAs (lanes D) were used to identify the bands corresponding to monomeric L RNAs. Lanes 3–6, protein to nucleotide molar ratios were 1:8, 1:4, 1:2, and 1:1. The monomeric and dimeric forms of L RNAs are indicated by m and d, respectively. The graphs were derived from the gels shown in this figure. Filled circles, Lwt RNA; open circles, LAU+1 RNA; open squares, LGC+1 RNA; open triangles, L+2 RNA; open diamonds, L+5 RNA.

Annealing of mini-TAR RNA to mini-TAR DNA hairpin in the presence of NCp7, NCp10, and NCp12 has been studied by gel shift assays [41]. The annealing data suggest that the nucleic acid chaperone activity decreases in the following order: NCp7 ~ NCp12 > NCp10. Here, we compared NCp7, NCp10, and NCp12 to promote dimerization of L3 RNA containing extensions of stem C (Figure 8 and Figure S1). L3 RNA dimerization induced by NCp7 decreased in the following order: Lwt ≥ LAU+1 > LGC+1 > L+2 > L+5 (Figure 8A). L+5 RNA did not form the extended dimer in the presence of increasing concentrations of NCp7 (Figure 8A, L+5). L3 RNA dimerization induced by NCp10 decreased in the following order: Lwt > LAU+1 > LGC+1 > L+2 > L+5 (Figure 8B). NCp10 was also unable to promote extended dimer formation when stem C was extended by five base pairs (Figure 8B, L+5).

The dimerization yields of Lwt and LAU+1 RNAs were similar in the presence of NCp7 and NCp12, whereas they were lower in the presence of NCp10 (Figure S1). The dimerization rates of LGC+1 RNA were not significantly different in the presence of NCp7 and NCp12. In contrast, NCp10 did not induce LGC+1 RNA dimerization as efficiently as NCp7 or NCp12. L+2 RNA displayed very low dimerization yields in the presence of the three nucleocapsid proteins at protein to nucleotide molar ratios lower than 1:1. At a protein to nucleotide molar ratio of 1:1, L+2 RNA dimerization was more efficient with NCp12 than with NCp7 or NCp10. The three nucleocapsid proteins were unable to promote extended dimer formation when stem C contained 10 base pairs (Figure S1, L+5).

## 4. Discussion

The L3 stem-loop is part of the highly structured 5′ leader sequence that has critical functions in virus replication, such as reverse transcription, translation, dimerization, and packaging [30]. The L3 stem-loop structure is conserved in alpharetroviruses and is involved in RNA dimerization in vitro [28,30,32]. In alpharetroviruses belonging to the type Schmidt-Ruppin A (SR-A), the primary importance of L3 in gRNA dimerization is supported by several studies [28,33,34,35]. In a previous study [35], we characterized the linkage structure of ALV (SR-A) RNA dimers induced by NCp12. We showed that the intermolecular base pairing involves loop C and stem C, but not stems A and B of L3 (Figure 2). Here, we performed a mutational analysis of an RNA transcript corresponding to L3 (SR-A) to determine the roles of stems and internal loops in the NCp12-mediated RNA dimerization process.

We first sought to evaluate the role of loop B in extended dimer formation. Interestingly, the m1LB and m2LB mutations did not lead to reductions in L3 RNA dimerization (Figure 4). These results show that conservation of the loop B sequence is not required for NCp12-mediated L3 dimerization. Consistent with this notion, the loop B sequence of the SR-A strain is different from that of the Prague C strain [32]. NCs from various retroviruses bind nucleic acids with a preference for sequences containing unpaired guanine residues [42,43,44,45]. To our knowledge, an extensive study of nucleic acid binding properties of NCp12 has not been performed. However, the three-dimensional structure of an RNA:NCp12 complex shows that an unpaired guanine residue can be important for NCp12 binding [46]. Both m1LB and m2LB mutants were designed, so that adenosine nucleobases replaced guanosine nucleobases in loop B (Figure 3). These mutants show that extended dimer formation does not require specific interactions between NCp12 and the guanine residues in loop B. Similarly, NCp7-mediated SL1 dimerization does not depend on guanine residues in the internal loop [27].

Structural rearrangements leading to extended dimer formation require destabilization of stem C by NCp12. Loop B appears to be an important destabilizing element in L3. Consistent with this view, the deletion of loop B dramatically reduced L3 RNA dimerization. However, this deletion did not abolish L3 RNA dimerization in the presence of NCp12 at high concentrations (mutant LdLB in Figure 4). This result indicates that NCp12 can destabilize the long stem containing the two bulges. As with NCp7 [47], NCp12 likely destabilizes the intramolecular base pairs surrounding bulges. Because the thermal stability of the LdLB dimer was increased by 13 °C compared to the wild-type dimer [35], the intermolecular base pairing in the LdLB extended dimer is not restricted to the loop C and stem C sequences. The deletion of loop A and the G bulge increases the length and stability of the L3 lower stem (mutant LdLA in Figure 3). LdLA RNA cannot dimerize (Figure 6) because the stability of its lower stem prevents the intermolecular base pairing involving loop C and stem C. These results are consistent with the observation that an alpharetrovirus mutant could replicate in the absence of both A and B stems [33], i.e., these stems are dispensable for gRNA dimerization. In addition, our results show that loop B does not allow sufficient destabilization of stem C by NCp12 in the LdLA mutant. Loop A and the G bulge are probably destabilizing elements in the NCp12-mediated L3 dimerization process. Interestingly, NCp7-mediated SL1 dimerization decreases by increasing the length of the lower stem of SL1 [27].

It is not surprising that NCp12 cannot destabilize the lower stem of LdLA that contains 14 base pairs without a bulge or loop interruption. Indeed, studies of HIV-1 RNA dimerization suggest that NCp7 can destabilize only short base-paired regions, i.e., fewer than 13 consecutive base pairs [21,26,27]. Moreover, Beltz et al. [47] showed that a mutant form of the cTAR DNA hairpin was barely destabilized by the NCp7(12–55) peptide (a truncated form of NCp7) because the lower stem contained 11 consecutive base pairs. The destabilizing effect of NCp7(12–55) and NCp7 on cTAR relies on the two bulges of the lower stem [47,48]. The length of stem C (five base pairs) is conserved in alpharetroviruses [28]. The maximum stem C length for efficient NCp12-mediated L3 dimerization likely corresponds to five base pairs. To test this hypothesis, the length of stem C was increased by one, two, or five base pairs (mutants LAU+1, LGC+1, L+2, and L+5 in Figure 3 and Figure 7). NCp12 was unable to promote extended dimer formation when stem C contained 10 base pairs. L3 dimerization did not occur efficiently when stem C contained seven base pairs. Furthermore, the extension of stem C by only one G:C base pair significantly decreased the dimerization yield at an NCp12 to nucleotide molar ratio lower than 1:2 (Figure 7, LGC+1). Therefore, five base pairs are the maximum stem C length for efficient NCp12-mediated L3 dimerization.

A previous study [41] compared the nucleic acid chaperone activity of HIV-1 NC (NCp7) to those of RSV (NCp12) and M-MuLV (NCp10) by analyzing the conversion of mini-TAR RNA and DNA hairpins into a heteroduplex. This study suggests that the chaperone activity decreases in the following order: NCp7 ~ NCp12 > NCp10. To date, the chaperone activities of the three NCs have not been compared by using an RNA dimerization assay. Because destabilization of stem C is necessary for extended dimer formation, NCp7 and NCp10 were tested for their ability to promote dimerization of L3 RNA containing extensions of stem C (Figure 8 and Figure S1). As with NCp12, NCp7 and NCp10 were unable to promote extended dimer formation when stem C contained 10 consecutive base pairs. In the presence of the three NCs, L3 dimerization did not occur efficiently when stem C possessed seven base pairs. Whatever the nucleocapsid protein, NC-mediated L3 dimerization decreased when stem C was extended by only one G:C base pair. However, the effect of this stem extension on NC-mediated L3 dimerization was more significant with NCp10 than with the other NCs. In contrast to NCp10, L3 dimerization induced by NCp7 or NCp12 was barely affected when stem C was extended by one A:U base pair. Formation of the extended dimer by the L3 wild type was less efficient with NCp10 than with both NCp7 and NCp12. Taken together, our results indicate that the nucleic acid chaperone activity decreases in the following order: NCp7 ~ NCp12 > NCp10. This ranking is consistent with the observation that NCp10 does not facilitate mini-TAR RNA/DNA annealing as efficiently as NCp7 or NCp12 [41]. Single-molecule DNA-stretching studies suggest that the reduced chaperone activity of NCp10 mainly results from the relatively slow kinetics of NCp10 dissociation from nucleic acids [41].

Our study is the first to show that an RNA stem-loop cannot be an NC-mediated RNA dimerization site if its stem contains 10 consecutive base pairs, thus favoring the notion that NC-mediated gRNA dimerization is regulated by the number of base pairing interactions. Stem-loop structures have been identified as RNA dimerization sites in various retroviruses [1]. Note that all these structures except HIV-1 SL1 possess a stem of fewer than seven consecutive base pairs. The mfold program [40] predicts almost the same stability for the upper part of SL1 (∆G = −6.7 kcal/mol) and L3 (∆G = −6.8 kcal/mol). Thus, it is not surprising that SL1 dimerization occurs efficiently in the presence of NCp7 [26,27], although the apical stem contains seven consecutive base pairs.

All retroviruses use specific host tRNAs as the primer for initiation of reverse transcription [49]. An 18 nt sequence in the viral RNA, termed the primer binding site (PBS), is complementary to the 3′ terminal 18 nt of the tRNA primer. The annealing of the tRNA primer to the viral RNA leads to an RNA duplex of 18 base pairs. In vitro studies showed that NCp7, NCp10, and NCp12 promote the annealing reaction [50,51]. An in vitro RNA structure-probing analysis supports the notion that HIV-1 Gag promotes partial annealing of tRNA^Lys,3^ to gRNA, followed by complete annealing by NCp7 [52]. Before the annealing step, both the PBS and the 3′ terminal 18 nt of primer are partially engaged in intramolecular base pairing. Destabilization of intramolecular base pairing is therefore required for PBS-tRNA duplex formation. Note that the intramolecular interactions do not exceed eight consecutive base pairs in the PBSs and tRNA primers of HIV-1, M-MuLV, and RSV [32,52,53,54,55,56,57]. Furthermore, an RNA helicase could play a role in promoting tRNA primer annealing [58,59].

The first strand transfer is an essential step of reverse transcription that requires base pairing of the repeat sequence (R) at the 3′-end of gRNA with the complementary r sequence at the 3′-end of minus-strand strong-stop DNA (ssDNA) [60]. Each complementary R/r sequence is partially engaged in an intramolecular base pairing that must be destabilized during the annealing reaction. In vitro studies suggest that NC facilitates the annealing reaction by destabilizing the DNA and RNA secondary structures (see Ref. [61] and references therein). Consistent with the notion that NC is an essential actor of the first-strand transfer, the intramolecular interactions do not exceed seven consecutive base pairs in the R regions of M-MuLV and RSV [32,55]. In contrast, the HIV-1 R region contains the TAR RNA stem-loop possessing 11 consecutive base pairs [62]. It is therefore surprising that NCp7 can destabilize this long stem. Destabilization of the TAR RNA hairpin by NCp7 has been investigated by combining single-molecule optical tweezers measurements with a quantitative mfold-based model [63]. This study suggests that NCp7 preferentially destabilizes four G-containing base pairs adjacent to defects in the secondary structure (two bulges and one G.U wobble base pair). Two of these G-containing base pairs are located in the long stem (11 base pairs) of the TAR RNA hairpin.

Taken together, these observations indicate that there are notable differences in the mechanism of NC-mediated annealing of DNA–RNA and RNA–RNA duplexes. A fully base-paired region of 10 base pairs cannot be an RNA dimerization site, whereas it can form a DNA–RNA duplex.

## Data Availability

Not applicable.

## Supplementary Materials

The following supporting information can be downloaded at: https://www.mdpi.com/article/10.3390/v14030606/s1, Figure S1: Influence of stem C extensions on L3 RNA dimerization induced by three different retroviral NCs.

## References

[1] Dubois N., Marquet R., Paillart J.-C., Bernacchi S. (2018). Retroviral RNA Dimerization: From Structure to Functions. Front. Microbiol..

[2] Onafuwa-Nuga A., Telesnitsky A. (2009). The Remarkable Frequency of Human Immunodeficiency Virus Type 1 Genetic Recombination. Microbiol. Mol. Biol. Rev..

[3] Pornillos O., Ganser-Pornillos B.K. (2019). Maturation of Retroviruses. Curr. Opin. Virol..

[4] Stoltzfus C.M., Snyder P.N. (1975). Structure of B77 Sarcoma Virus RNA: Stabilization of RNA after Packaging. J. Virol..

[5] Fu W., Rein A. (1993). Maturation of Dimeric Viral RNA of Moloney Murine Leukemia Virus. J. Virol..

[6] Fu W., Gorelick R.J., Rein A. (1994). Characterization of Human Immunodeficiency Virus Type 1 Dimeric RNA from Wild-Type and Protease-Defective Virions. J. Virol..

[7] Stewart L., Schatz G., Vogt V.M. (1990). Properties of Avian Retrovirus Particles Defective in Viral Protease. J. Virol..

[8] Shehu-Xhilaga M., Kraeusslich H.G., Pettit S., Swanstrom R., Lee J.Y., Marshall J.A., Crowe S.M., Mak J. (2001). Proteolytic Processing of the P2/Nucleocapsid Cleavage Site Is Critical for Human Immunodeficiency Virus Type 1 RNA Dimer Maturation. J. Virol..

[9] Ohishi M., Nakano T., Sakuragi S., Shioda T., Sano K., Sakuragi J. (2011). The Relationship between HIV-1 Genome RNA Dimerization, Virion Maturation and Infectivity. Nucleic Acids Res..

[10] Méric C., Spahr P.F. (1986). Rous Sarcoma Virus Nucleic Acid-Binding Protein P12 Is Necessary for Viral 70S RNA Dimer Formation and Packaging. J. Virol..

[11] Méric C., Gouilloud E., Spahr P.F. (1988). Mutations in Rous Sarcoma Virus Nucleocapsid Protein P12 (NC): Deletions of Cys-His Boxes. J. Virol..

[12] Bieth E., Gabus C., Darlix J.L. (1990). A Study of the Dimer Formation of Rous Sarcoma Virus RNA and of Its Effect on Viral Protein Synthesis in Vitro. Nucleic Acids Res..

[13] Darlix J.L., Gabus C., Nugeyre M.T., Clavel F., Barré-Sinoussi F. (1990). Cis Elements and Trans-Acting Factors Involved in the RNA Dimerization of the Human Immunodeficiency Virus HIV-1. J. Mol. Biol..

[14] Prats A.C., Roy C., Wang P.A., Erard M., Housset V., Gabus C., Paoletti C., Darlix J.L. (1990). Cis Elements and Trans-Acting Factors Involved in Dimer Formation of Murine Leukemia Virus RNA. J. Virol..

[15] Laughrea M., Jetté L. (1994). A 19-Nucleotide Sequence Upstream of the 5’ Major Splice Donor Is Part of the Dimerization Domain of Human Immunodeficiency Virus 1 Genomic RNA. Biochemistry.

[16] Muriaux D., Fossé P., Paoletti J. (1996). A Kissing Complex Together with a Stable Dimer Is Involved in the HIV-1Lai RNA Dimerization Process in Vitro. Biochemistry.

[17] Skripkin E., Paillart J.C., Marquet R., Ehresmann B., Ehresmann C. (1994). Identification of the Primary Site of the Human Immunodeficiency Virus Type 1 RNA Dimerization in Vitro. Proc. Natl. Acad. Sci. USA.

[18] Laughrea M., Jetté L. (1996). Kissing-Loop Model of HIV-1 Genome Dimerization: HIV-1 RNAs Can Assume Alternative Dimeric Forms, and All Sequences Upstream or Downstream of Hairpin 248-271 Are Dispensable for Dimer Formation. Biochemistry.

[19] Paillart J.C., Skripkin E., Ehresmann B., Ehresmann C., Marquet R. (1996). A Loop-Loop “Kissing” Complex Is the Essential Part of the Dimer Linkage of Genomic HIV-1 RNA. Proc. Natl. Acad. Sci. USA.

[20] Muriaux D., De Rocquigny H., Roques B.P., Paoletti J. (1996). NCp7 Activates HIV-1Lai RNA Dimerization by Converting a Transient Loop-Loop Complex into a Stable Dimer. J. Biol. Chem..

[21] Andersen E.S., Contera S.A., Knudsen B., Damgaard C.K., Besenbacher F., Kjems J. (2004). Role of the Trans-Activation Response Element in Dimerization of HIV-1 RNA. J. Biol. Chem..

[22] Windbichler N., Werner M., Schroeder R. (2003). Kissing Complex-Mediated Dimerisation of HIV-1 RNA: Coupling Extended Duplex Formation to Ribozyme Cleavage. Nucleic Acids Res..

[23] Ulyanov N.B., Mujeeb A., Du Z., Tonelli M., Parslow T.G., James T.L. (2006). NMR Structure of the Full-Length Linear Dimer of Stem-Loop-1 RNA in the HIV-1 Dimer Initiation Site. J. Biol. Chem..

[24] Keane S.C., Van V., Frank H.M., Sciandra C.A., McCowin S., Santos J., Heng X., Summers M.F. (2016). NMR Detection of Intermolecular Interaction Sites in the Dimeric 5’-Leader of the HIV-1 Genome. Proc. Natl. Acad. Sci. USA.

[25] Blakemore R.J., Burnett C., Swanson C., Kharytonchyk S., Telesnitsky A., Munro J.B. (2021). Stability and Conformation of the Dimeric HIV-1 Genomic RNA 5’UTR. Biophys. J..

[26] Takahashi K.I., Baba S., Chattopadhyay P., Koyanagi Y., Yamamoto N., Takaku H., Kawai G. (2000). Structural Requirement for the Two-Step Dimerization of Human Immunodeficiency Virus Type 1 Genome. RNA.

[27] Mujeeb A., Ulyanov N.B., Georgantis S., Smirnov I., Chung J., Parslow T.G., James T.L. (2007). Nucleocapsid Protein-Mediated Maturation of Dimer Initiation Complex of Full-Length SL1 Stemloop of HIV-1: Sequence Effects and Mechanism of RNA Refolding. Nucleic Acids Res..

[28] Fossé P., Motté N., Roumier A., Gabus C., Muriaux D., Darlix J.L., Paoletti J. (1996). A Short Autocomplementary Sequence Plays an Essential Role in Avian Sarcoma-Leukosis Virus RNA Dimerization. Biochemistry.

[29] Polge E., Darlix J.L., Paoletti J., Fossé P. (2000). Characterization of Loose and Tight Dimer Forms of Avian Leukosis Virus RNA. J. Mol. Biol..

[30] Hackett P.B., Dalton M.W., Johnson D.P., Petersen R.B. (1991). Phylogenetic and Physical Analysis of the 5’ Leader RNA Sequences of Avian Retroviruses. Nucleic Acids Res..

[31] Banks J.D., Linial M.L. (2000). Secondary Structure Analysis of a Minimal Avian Leukosis-Sarcoma Virus Packaging Signal. J. Virol..

[32] Liu S., Maldonado R.K., Rye-McCurdy T., Binkley C., Bah A., Chen E.C., Rice B.L., Parent L.J., Musier-Forsyth K. (2020). Rous Sarcoma Virus Genomic RNA Dimerization Capability In Vitro Is Not a Prerequisite for Viral Infectivity. Viruses.

[33] Doria-Rose N.A., Vogt V.M. (1998). In Vivo Selection of Rous Sarcoma Virus Mutants with Randomized Sequences in the Packaging Signal. J. Virol..

[34] Chen E.C., Maldonado R.J.K., Parent L.J. (2021). Visualizing Rous Sarcoma Virus Genomic RNA Dimerization in the Nucleus, Cytoplasm, and at the Plasma Membrane. Viruses.

[35] Ben Ali M., Chaminade F., Kanevsky I., Ennifar E., Josset L., Ficheux D., Darlix J.-L., Fossé P. (2007). Structural Requirements for Nucleocapsid Protein-Mediated Dimerization of Avian Leukosis Virus RNA. J. Mol. Biol..

[36] Cornille F., Mely Y., Ficheux D., Savignol I., Gerard D., Darlix J.L., Fournie-Zaluski M.C., Roques B.P. (1990). Solid Phase Synthesis of the Retroviral Nucleocapsid Protein NCp10 of Moloney Murine Leukaemia Virus and Related “Zinc-Fingers” in Free SH Forms. Influence of Zinc Chelation on Structural and Biochemical Properties. Int. J. Pept. Protein Res..

[37] De Rocquigny H., Ficheux D., Gabus C., Fournié-Zaluski M.C., Darlix J.L., Roques B.P. (1991). First Large Scale Chemical Synthesis of the 72 Amino Acid HIV-1 Nucleocapsid Protein NCp7 in an Active Form. Biochem. Biophys. Res. Commun..

[38] Chen Y., Maskri O., Chaminade F., René B., Benkaroun J., Godet J., Mély Y., Mauffret O., Fossé P. (2016). Structural Insights into the HIV-1 Minus-Strand Strong-Stop DNA. J. Biol. Chem..

[39] Schwartz D.E., Tizard R., Gilbert W. (1983). Nucleotide Sequence of Rous Sarcoma Virus. Cell.

[40] Zuker M. (2003). Mfold Web Server for Nucleic Acid Folding and Hybridization Prediction. Nucleic Acids Res..

[41] Stewart-Maynard K.M., Cruceanu M., Wang F., Vo M.-N., Gorelick R.J., Williams M.C., Rouzina I., Musier-Forsyth K. (2008). Retroviral Nucleocapsid Proteins Display Nonequivalent Levels of Nucleic Acid Chaperone Activity. J. Virol..

[42] Fisher R.J., Rein A., Fivash M., Urbaneja M.A., Casas-Finet J.R., Medaglia M., Henderson L.E. (1998). Sequence-Specific Binding of Human Immunodeficiency Virus Type 1 Nucleocapsid Protein to Short Oligonucleotides. J. Virol..

[43] Urbaneja M.A., McGrath C.F., Kane B.P., Henderson L.E., Casas-Finet J.R. (2000). Nucleic Acid Binding Properties of the Simian Immunodeficiency Virus Nucleocapsid Protein NCp8. J. Biol. Chem..

[44] Morcock D.R., Katakam S., Kane B.P., Casas-Finet J.R. (2002). Fluorescence and Nucleic Acid Binding Properties of Bovine Leukemia Virus Nucleocapsid Protein. Biophys. Chem..

[45] Dey A., York D., Smalls-Mantey A., Summers M.F. (2005). Composition and Sequence-Dependent Binding of RNA to the Nucleocapsid Protein of Moloney Murine Leukemia Virus. Biochemistry.

[46] Zhou J., Bean R.L., Vogt V.M., Summers M. (2007). Solution Structure of the Rous Sarcoma Virus Nucleocapsid Protein: MuPsi RNA Packaging Signal Complex. J. Mol. Biol..

[47] Beltz H., Azoulay J., Bernacchi S., Clamme J.-P., Ficheux D., Roques B., Darlix J.-L., Mély Y. (2003). Impact of the Terminal Bulges of HIV-1 cTAR DNA on Its Stability and the Destabilizing Activity of the Nucleocapsid Protein NCp7. J. Mol. Biol..

[48] Cosa G., Harbron E.J., Zeng Y., Liu H.-W., O’Connor D.B., Eta-Hosokawa C., Musier-Forsyth K., Barbara P.F. (2004). Secondary Structure and Secondary Structure Dynamics of DNA Hairpins Complexed with HIV-1 NC Protein. Biophys. J..

[49] Marquet R., Isel C., Ehresmann C., Ehresmann B. (1995). tRNAs as Primer of Reverse Transcriptases. Biochimie.

[50] Prats A.C., Sarih L., Gabus C., Litvak S., Keith G., Darlix J.L. (1988). Small Finger Protein of Avian and Murine Retroviruses Has Nucleic Acid Annealing Activity and Positions the Replication Primer tRNA onto Genomic RNA. EMBO J..

[51] Barat C., Lullien V., Schatz O., Keith G., Nugeyre M.T., Grüninger-Leitch F., Barré-Sinoussi F., LeGrice S.F., Darlix J.L. (1989). HIV-1 Reverse Transcriptase Specifically Interacts with the Anticodon Domain of Its Cognate Primer tRNA. EMBO J..

[52] Seif E., Niu M., Kleiman L. (2015). In Virio SHAPE Analysis of tRNA(Lys3) Annealing to HIV-1 Genomic RNA in Wild Type and Protease-Deficient Virus. Retrovirology.

[53] Keith G., Heyman T. (1990). Heterogeneities in Vertebrate tRNAs(Trp) Avian Retroviruses Package Only as a Primer the tRNA(Trp) Lacking Modified M2G in Position 7. Nucleic Acids Res..

[54] Baudin F., Marquet R., Isel C., Darlix J.L., Ehresmann B., Ehresmann C. (1993). Functional Sites in the 5’ Region of Human Immunodeficiency Virus Type 1 RNA Form Defined Structural Domains. J. Mol. Biol..

[55] Mougel M., Tounekti N., Darlix J.L., Paoletti J., Ehresmann B., Ehresmann C. (1993). Conformational Analysis of the 5’ Leader and the Gag Initiation Site of Mo-MuLV RNA and Allosteric Transitions Induced by Dimerization. Nucleic Acids Res..

[56] Isel C., Ehresmann C., Keith G., Ehresmann B., Marquet R. (1995). Initiation of Reverse Transcription of HIV-1: Secondary Structure of the HIV-1 RNA/tRNA(3Lys) (Template/Primer). J. Mol. Biol..

[57] Fossé P., Mougel M., Keith G., Westhof E., Ehresmann B., Ehresmann C. (1998). Modified Nucleotides of tRNAPro Restrict Interactions in the Binary Primer/Template Complex of M-MuLV. J. Mol. Biol..

[58] Xing L., Liang C., Kleiman L. (2011). Coordinate Roles of Gag and RNA Helicase A in Promoting the Annealing of tRNALys,3 to HIV-1 RNA. J. Virol..

[59] Xing L., Niu M., Kleiman L. (2012). In Vitro and in Vivo Analysis of the Interaction between RNA Helicase A and HIV-1 RNA. J. Virol..

[60] Gilboa E., Mitra S.W., Goff S., Baltimore D. (1979). A Detailed Model of Reverse Transcription and Tests of Crucial Aspects. Cell.

[61] René B., Mauffret O., Fossé P. (2018). Retroviral Nucleocapsid Proteins and DNA Strand Transfers. Biochim. Open.

[62] Watts J.M., Dang K.K., Gorelick R.J., Leonard C.W., Bess J.W., Swanstrom R., Burch C.L., Weeks K.M. (2009). Architecture and Secondary Structure of an Entire HIV-1 RNA Genome. Nature.

[63] McCauley M.J., Rouzina I., Manthei K.A., Gorelick R.J., Musier-Forsyth K., Williams M.C. (2015). Targeted Binding of Nucleocapsid Protein Transforms the Folding Landscape of HIV-1 TAR RNA. Proc. Natl. Acad. Sci. USA.

