# Supplementary material for: RNA Structural Requirements for Nucleocapsid Protein-Mediated Extended Dimer Formation
Source: Viruses. 2022 Mar 15;14(3):606. doi: 10.3390/v14030606 (PMC8953772; doi:10.3390/v14030606)
Supplement: Supplementary file 1 [file viruses-14-00606-s001.zip › viruses-1629186-supplementary.pdf]

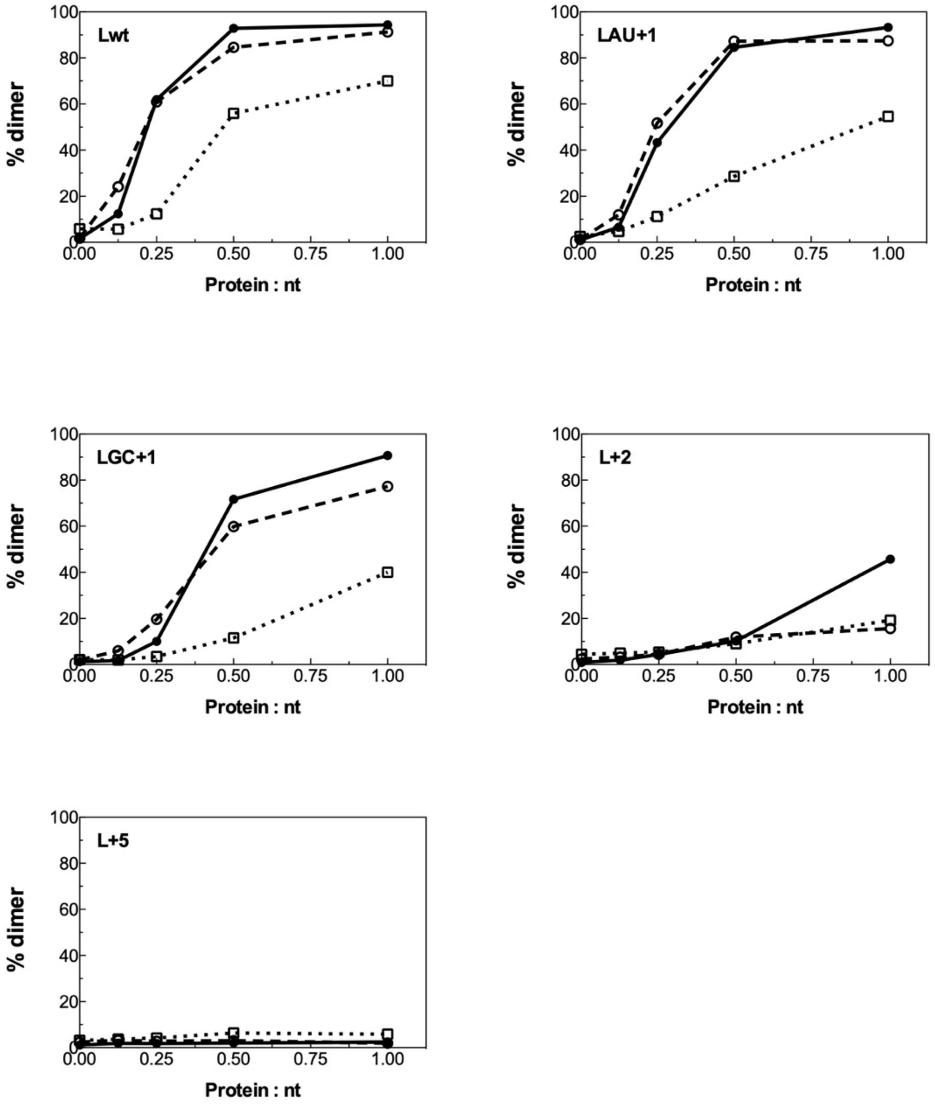

Figure S1. Influence of stem C extensions on L3 RNA dimerization induced by three different retroviral NCs. Data are derived from experiments shown in Figures 7 and 8. Filled circles, NCp12; open circles, NCp7; open squares, NCp10.
